# MicroRNA-144-3p protects against chemotherapy-induced apoptosis of ovarian granulosa cells and activation of primordial follicles by targeting MAP3K9

**DOI:** 10.1186/s40001-023-01231-2

**Published:** 2023-08-03

**Authors:** Meng Liu, Bang Xiao, Yiqing Zhu, Meiting Chen, Jinfeng Huang, Haiyan Guo, Fang Wang

**Affiliations:** 1 Department of Medical Genetics, Naval Medical University, Shanghai, 200433 China; 2 Department of Assisted Reproduction, Shanghai Ninth People’s Hospital, Shanghai Jiaotong University School of Medicine, Shanghai, 200011 China

## Abstract

Premature ovarian failure (POF) is defined by amenorrhea, ovarian atrophy, hypoestrogenism, elevated gonadotropin level, and infertility under the age of 40. POF is frequently induced by chemotherapeutic agents. However, the underlying mechanisms regarding chemotherapy-mediated damage to ovarian function are unclear. In this study, enhanced apoptosis of granulosa cells (GCs) and aberrant activation of primordial follicles were observed in a POF mouse model induced by cisplatin. We subsequently observed significant downregulation of miR-144-3p and upregulation of mitogen-activated protein kinase kinase kinase 9 (MAP3K9) in primary ovarian GCs from POF mice, as revealed by microarrays. Furthermore, *MAP3K9* expression was higher in human ovarian granulosa cells (COV434) treated with cisplatin and was identified as a novel target of miR-144-3p. Functional analysis revealed that miR-144-3p attenuated cisplatin induced apoptosis of GCs via silencing *MAP3K9* expression, which suppressed the activity of the downstream p38 mitogen activated protein kinase (MAPK) pathway. Meanwhile, miR-144-3p prevented premature primordial follicle depletion in cisplatin-induced POF mice through targeting *Map3k9*, which led to a decline in the phosphorylation and activation of the phosphatidylinositol-4,5-bisphosphate 3-kinase (PI3K)/protein kinase b (AKT) pathway. Taken together, this study revealed the protective effects of miR-144-3p on ovarian function and shed light on the epigenetic regulatory mechanism in the development of POF, which might provide new biomarkers for the ovarian reserve.

**Supplementary Information:**

The online version contains supplementary material available at 10.1186/s40001-023-01231-2.

## Introduction

Premature ovarian failure (POF), also known as premature ovarian insufficiency (POI), is a subclass of endocrine and reproductive disorders characterized by amenorrhea, increased gonadotropin level, estrogen deficiency, ovarian atrophy, decreased sexual activity, and decreased fertility in women under the age of 40, which affects approximately 1% of women in the general population [1, 2]. However, the etiology of POF is unclear. Among the underlying mechanisms, destruction of primordial follicles by toxic agents, activation of proapoptotic signaling pathways, or accelerated follicular recruitment might lead to premature exhaustion of the primordial follicle pool or disturbances of follicle function and accelerated premature follicle depletion, which have been demonstrated to be related to follicular atresia and POF [3–6]. Exploring the association between most of these pathogenic factors and their role in ovarian function is necessary to determine the mechanisms responsible for the heterogeneity of POF.

MicroRNAs (miRNAs) are a class of small (18–22 nucleotides) endogenous noncoding RNAs that can negatively regulate gene expression via post-transcriptional gene silencing [7, 8]. It has been proposed that aberrant expression and dysfunction of miRNAs might be involved in physiological processes and human diseases, such as the events of mammalian reproduction [9–11]. Accumulating evidence shows that deregulation of miRNAs is closely related to ovarian function, including granulosa cell (GC) apoptosis, oocyte maturation, oocyte apoptosis, recruitment of primordial follicles, and localization of migrating primordial germ cells [9, 12–15]. However, the contribution of miRNAs in the pathogenesis of POF has yet to be determined. Therefore, the aim of the present study was to investigate differentially expressed miRNAs in a mouse POF model and reveal the association between miRNAs and diminished ovarian function, which would help to unveil the possible molecular mechanisms underlying the pathogenesis of POF.

Herein, we established a cisplatin-induced mouse POF model to determine whether cisplatin affects GC apoptosis and primordial follicle activation in the ovary. Next, we analyzed miRNA and messenger RNA (mRNA) profiles and noted that miR-144-3p was significantly downregulated in GCs obtained from cisplatin-induced POF mice. Bioinformatic analysis combined with in vitro and in vivo experiments indicated that miR-144-3p alleviated GC apoptosis and ovarian primordial follicle loss induced by cisplatin by directly targeting the mRNA encoding mitogen-activated protein kinase kinase kinase 9 (MAP3K9). Furthermore, the detailed underlying molecular mechanisms were explored. We demonstrated that miR-144-3p could serve as a potential biomarker for ovarian reserve evaluation.

## Materials and methods

### Animal experiments

All animal experiments were approved by the Committee on Ethics of Biomedicine Research, Naval Medical University. Female C57BL/6 mice (8 weeks) with normal 4–5-day estrous cycles were used in all experiments described below.

### Establishment of the premature ovarian failure mouse model

To establish the chemotherapy-induced POF model, 8-week-old female C57BL/6 mice provided by the laboratory animal center of Naval Medical University were administered with 2 mg/kg cisplatin (Sigma-Aldrich, St. Louis, MO, USA) daily via intraperitoneal injection for 7 days. Mice in the control group were injected intraperitoneally with an equal amount of physiological saline. The estrous cycles were routinely assessed by vaginal smear, and venous blood samples were collected in the diestrus stage after 7 days of treatment.

### Enzyme-linked immunosorbent assay (ELISA)

Blood samples of each mouse were collected and centrifuged at 3220×*g* for 15 min. The serum levels of estradiol (E_2_) and follicle stimulating hormone (FSH) were measured using ELISA kits (Mlbio, Shanghai, China) according to the manufacturer’s instructions.

### Ovarian follicle counting

Seven days after cisplatin treatment, the mice were euthanized, and the ovaries were harvested and fixed using 4% formaldehyde for 24 h, embedded in paraffin, serially sectioned at 5 μm, and mounted on every fifth section. The sections were then stained with hematoxylin and erosion (H&E) for further histological examination. The number of ovarian primordial, primary, secondary, antral and atretic follicles was counted under a light microscope according to the accepted definitions described previously [16].

### Terminal deoxynucleotidyl transferase-mediated dUTP–biotin nick end labeling (TUNEL) assay

An In situ Cell Death Detection Kit (Roche, Germany) was used to detect GCs apoptosis in mouse ovarian tissue sections according to the manufacturer’s instruction. The sections were observed with a fluorescence microscope, and the number of TUNEL-positive GCs was counted.

### Isolation of mouse primary ovarian GCs and cell culture

Mouse primary ovarian GCs were isolated from female C57BL/6 mice. The female mice were injected intraperitoneally with pregnant mare serum gonadotropin (PMSG, Solarbio, Beijing, China). After 48 h, bilateral ovaries were isolated mechanically. GCs were obtained under a stereomicroscope and washed with phosphate buffered saline (PBS) three times. The GCs were then collected by brief centrifugation and cultured in Dulbecco’s modified Eagle’s medium (DMEM)/F12 (HyClone, Logan, UT, USA) medium supplemented with 10% fetal bovine serum (Biological Industries, ISV, Kibbutz Beit-Haemek, Israel), 100 U/ml streptomycin and 100 U/ml penicillin (Invitrogen, Waltham, MA, USA). GCs were identified using anti-follicle stimulating hormone receptor (FSHR) antibodies (Abcam, Cambridge, UK). The COV434 cell line (human ovarian GCs) was cultured in DMEM/F12 containing 10% fetal bovine serum and 1% penicillin–streptomycin. All cells were cultured at 37 ℃ in a humidified atmosphere of 5% CO_2_.

### Microarray analysis

GCs from the control and POF groups were used for miRNA and mRNA expression profiling assay. RNA quantity and quality were measured by NanoDrop ND-1000, and RNA integrity was assessed by standard denaturing agarose gel electrophoresis. Sample labeling and array hybridization were performed according to the Agilent miRNA Microarray System with miRNA Complete Labeling and Hyb Kit protocol (Agilent Technology). The differentially expressed genes among the different groups were determined by KEGG and GO analyses. Microarray profiling and data analyses were performed by KangChen Bio-tech, Shanghai, China.

### RNA isolation and quantitative real-time reverse transcription PCR (qRT-PCR)

Total RNA from cultured cells was isolated using the TRIzol reagent (Invitrogen). cDNA was synthesized from purified total RNA (1 μg) using a PrimeScript RT reagent kit (Takara, Dalian, China) according to the manufacturer’s instructions. The quantitative real-time PCR (qPCR) step of the qRT-PCR protocol was performed using SYBR Green Real-time PCR Master Mix (Takara) and a Real-time PCR system (Applied Biosystems, Foster City, CA, USA). U6 and *ACTB* (encoding β-actin) were used as internal controls for miRNAs and mRNAs, respectively. Fold change was calculated according to the 2^−ΔΔCt^ method, and data are represented relative to the expression in control cells. The specific primers are listed in Additional file 1: Table S1.

### Western blotting analysis

Total proteins from cells were prepared, separated by sodium dodecyl sulfate–polyacrylamide gel electrophoresis, and transferred onto polyvinylidene fluoride membranes (Millipore, Billerica, MA, USA). After blocking with 5% milk in Tris-buffered saline Tween 20 (TBST) for 1 h at room temperature, the membranes were incubated with primary antibodies at 4 ℃ overnight. Then, the membranes were incubated with IRdye 800-conjugated goat anti-rabbit IgG and/or IRdye 700-conjugated goat anti-mouse IgG for 1 h at room temperature. An Odyssey infrared scanner (Li-COR Biosciences, Lincoln, NE, USA) was used to visualize the immunoreactive protein bands. The relative intensity of the protein bands was expressed relative to that of the control. The levels of β-tubulin or β-actin were used as internal standards. The following primary antibodies were used: anti-MAP3K9 (Cell Signaling Technology, Danvers, MA, USA; 5029, 1:1000), anti-Caspase-3 (Cell Signaling Technology, 9662, 1:1000), anti-general transcription factor IIH subunit 2 (p44)/proteasome 26S subunit, ATPase 6 (p42) (Cell Signaling Technology, 4695, 1:1000), anti-mitogen-activated protein kinase 14 (MAPK14, or p38) (Cell Signaling Technology, 8690, 1:1000), anti-phosphorylated (p)44/42 (Cell Signaling Technology, 4370, 1:1000), anti-p–p38 (Cell Signaling Technology, 4511, 1:1000), anti-protein kinase B (AKT) (Proteintech, Rosemont, IL, USA;10176-2-AP, 1:1000), anti-p-AKT (Proteintech, 66444-1-Ig, 1:2000), anti-forkhead box O3 (FoxO3A) (Signalway Antibody, Greenbelt, MD, USA; 40937, 1:1000), anti-p-FoxO3A (Signalway Antibody, 12199, 1:1000), anti-β-actin (Proteintech, 66009-1-Ig, 1:5000), and anti-β-tubulin (Proteintech, 10094-1-AP, 1:5000).

### Cell transfection

COV434 cells were transfected with miR-144-3p agomir, antagomir, specific small interfering RNAs (siRNAs) for MAP3K9, or negative control (agomir NC, antagomir NC, siNC) (GenePharma, Shanghai, China) using Lipofectamine 3000 (Invitrogen) according to the manufacturer’s instructions. After 24 h of transfection, the cells were collected for qRT-PCR and western blotting analysis.

### Luciferase reporter assay

A luciferase reporter plasmid containing the wild-type 3′ untranslated region (UTR) of *MAP3K9* was constructed and the *MAP3K9 3*′ UTR containing the mutated has miR-144-3p binding site was cloned into the same reporter plasmid. The above luciferase reporter plasmids were co-transfected with miR-144-3p mimics or negative control (NC) into COV434 cells cultured in 6-well plates using Lipofectamine 3000 (Invitrogen). Subsequently, the luciferase activity of the cell lysates was measured 48 h later using a Dual-Glo Luciferase Assay kit (Promega, Madison, WI, USA) according to the manufacturer’s instructions.

### In vivo experiments

The POF mice were created as in 'Establishment of the premature ovarian failure mouse model'. After 7 days of treatment, 20 μl of the miR-144-3p agomir or controls at 1 nmol, with Lipofectamine 3000 (Invitrogen), were injected into the left and right ovarian bursa using insulin syringes, respectively. At 48 h after injection, the ovaries were collected for western blotting assessment and histological examination.

### Statistical analysis

All experiments were performed three times. Data are expressed as the mean ± standard deviation (SD) and were analyzed using Student’s *t* test when the data were normally distributed, using GraphPad Prism 6 software (GraphPad Inc., La Jolla, CA, USA). When the data were not normally distributed, a nonparametric test was applied. The significance of differences among groups was assessed using one-way analysis of variance (ANOVA). Statistical significance was defined as *P* < 0.05.

## Results

### Evaluation of the cisplatin-induced POF mouse model

**Fig. 1:**
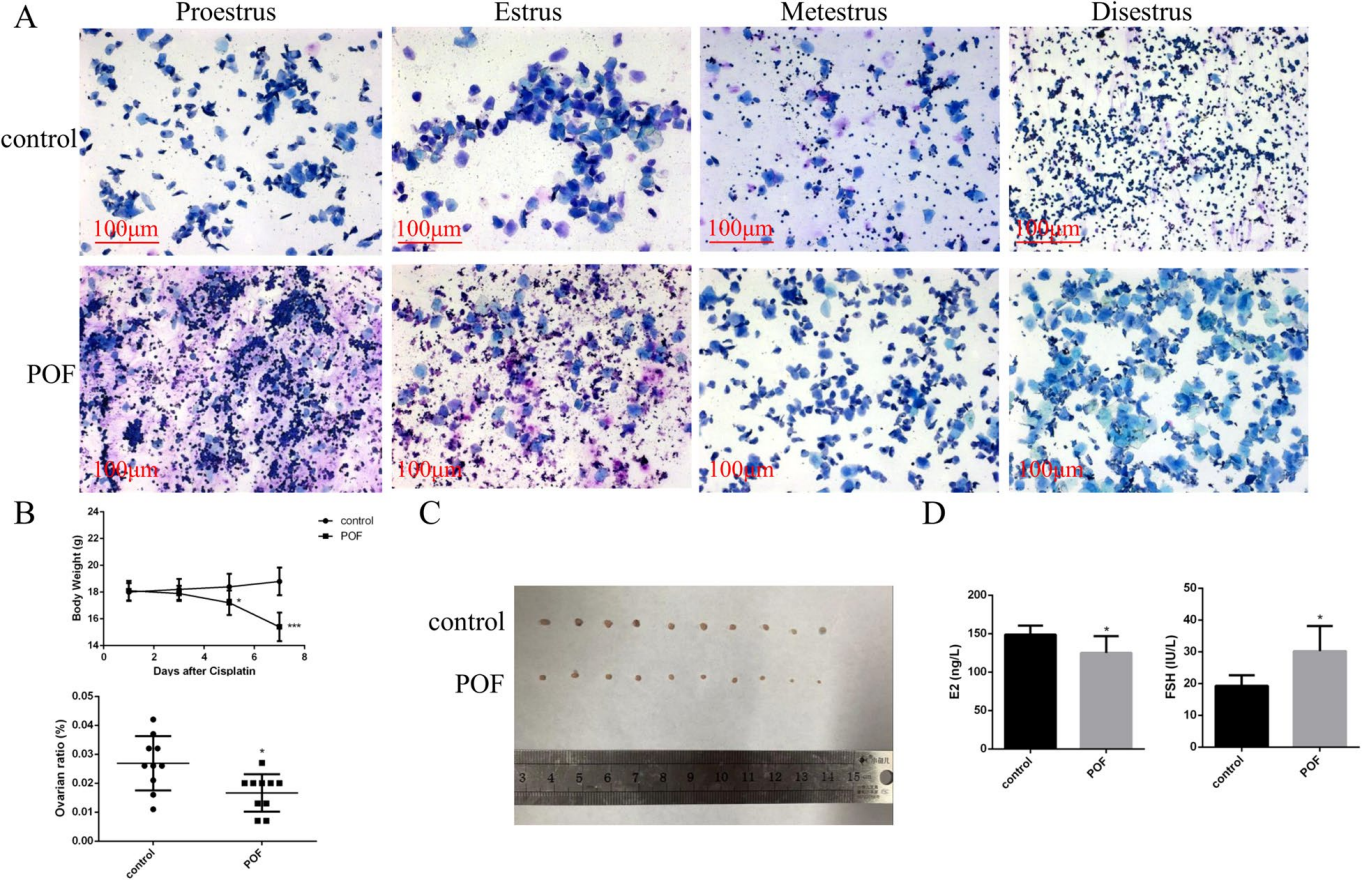
Cisplatin-induced POF mouse model. **A** Vaginal exfoliative cell smear from normal mice and POF mice. **B** Bar graph illustrating the body weight of mice with or without cisplatin treatment. **C** Comparison of the ovarian size and weight ratio between the control and POF groups. **D** Serum concentration of E_2_ and FSH determined by ELISA in the control and POF groups. Control group, *n* = 10; POF group, *n* = 10. POF: premature ovarian failure; E_2_: estradiol; FSH: follicle-stimulating hormone; ELISA: enzyme-linked immunosorbent assay. *P < 0.05; ***P < 0.001, two-tailed Student’s *t* test. Scale bar, 100 μm

Animals were weighed before treatment with or without cisplatin at 2 mg/kg. To ensure the successful establishment of POF mouse model, we evaluated several ovarian function parameters and the estrous cycles at 7 days after cisplatin treatment. Regular estrous cycles were observed in the control group and lasted 4–6 days. In contrast, significantly irregular estrous cycles were observed in the mice receiving cisplatin, which exhibited a shorter proestrus and estrus, and a longer diestrus (Fig. 1A). Then, we evaluated the body weight of mice following chemotherapy and observed significant weight loss, which began on day 5 in the POF group, compared with that in the control group (Fig. 1B). We also weighed the ovaries at 7 days after cisplatin treatment and found that the ratio of ovarian weight obtained from the POF group was significantly lower than that obtained from the control group (Fig. 1B). In addition, Fig. 1C shows the different ovarian sizes across the two groups. Plasma samples were collected to measure the sex hormone levels. As shown in Fig. 1D, following cisplatin treatment the level of E_2_ decreased markedly, while the FSH level in the cisplatin-treated group was significantly increased compared with that in the control group.

### Effects of cisplatin treatment on apoptosis of ovarian GCs in mice

**Fig. 2:**
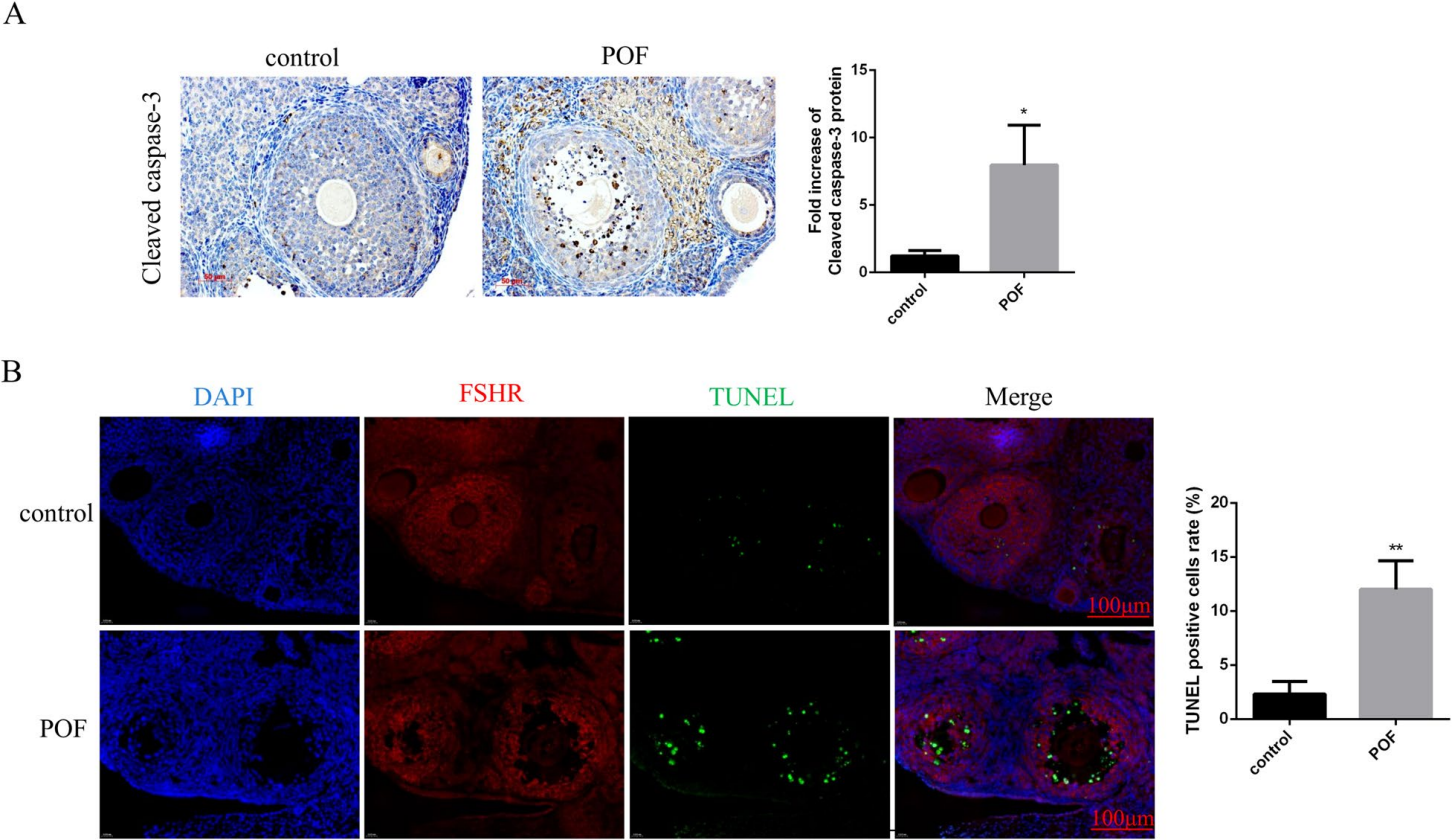
Effects of cisplatin treatment on apoptosis of GCs in mice. **A** Cleaved caspase-3 staining was analyzed by immunohistochemistry in the control and POF groups. Cleaved caspase-3 positive cells are shown as brown with the cell nucleus stained blue. **B** Apoptosis of GCs in ovarian tissues examined by TUNEL analysis. The red staining indicates FSHR-positive GCs. The green stain indicates TUNEL-positive GCs. The blue DAPI stain indicates the cell nucleus. Control group, *n* = 10; POF group, *n* = 10. GCs: granulosa cells; POF: premature ovarian failure; TUNEL: terminal deoxynucleotidyl transferase-mediated dUTP–biotin nick end labeling; DAPI: 4′6-diamidino-2-phenylindole. *P < 0.05; **P < 0.01, two-tailed Student’s *t* test. Scale bars represent 50 μm (**A**), and 100 μm (**B**)

Apoptosis of GCs is the key mechanism responsible for the development of follicular atresia and follicle loss. To evaluate the effect of cisplatin on GC apoptosis, we detected the protein level of cleaved caspase-3 in the injured ovaries by immunohistochemistry analysis. The result revealed that cleaved caspase-3 levels were significantly increased in the ovaries of POF mice compared with those in the control group (Fig. 2A). Furthermore, we observed that the number of TUNEL and FSHR (follicle stimulating hormone receptor, GC-specific marker) double-positive cells were clearly increased in the ovarian section of the POF group compared with that in the control group (Fig. 2B). The data suggest that GCs apoptosis occurs during the development of ovarian function failure in POF mice.

### Effects of cisplatin treatment on ovarian follicle activation in mice

**Fig. 3:**
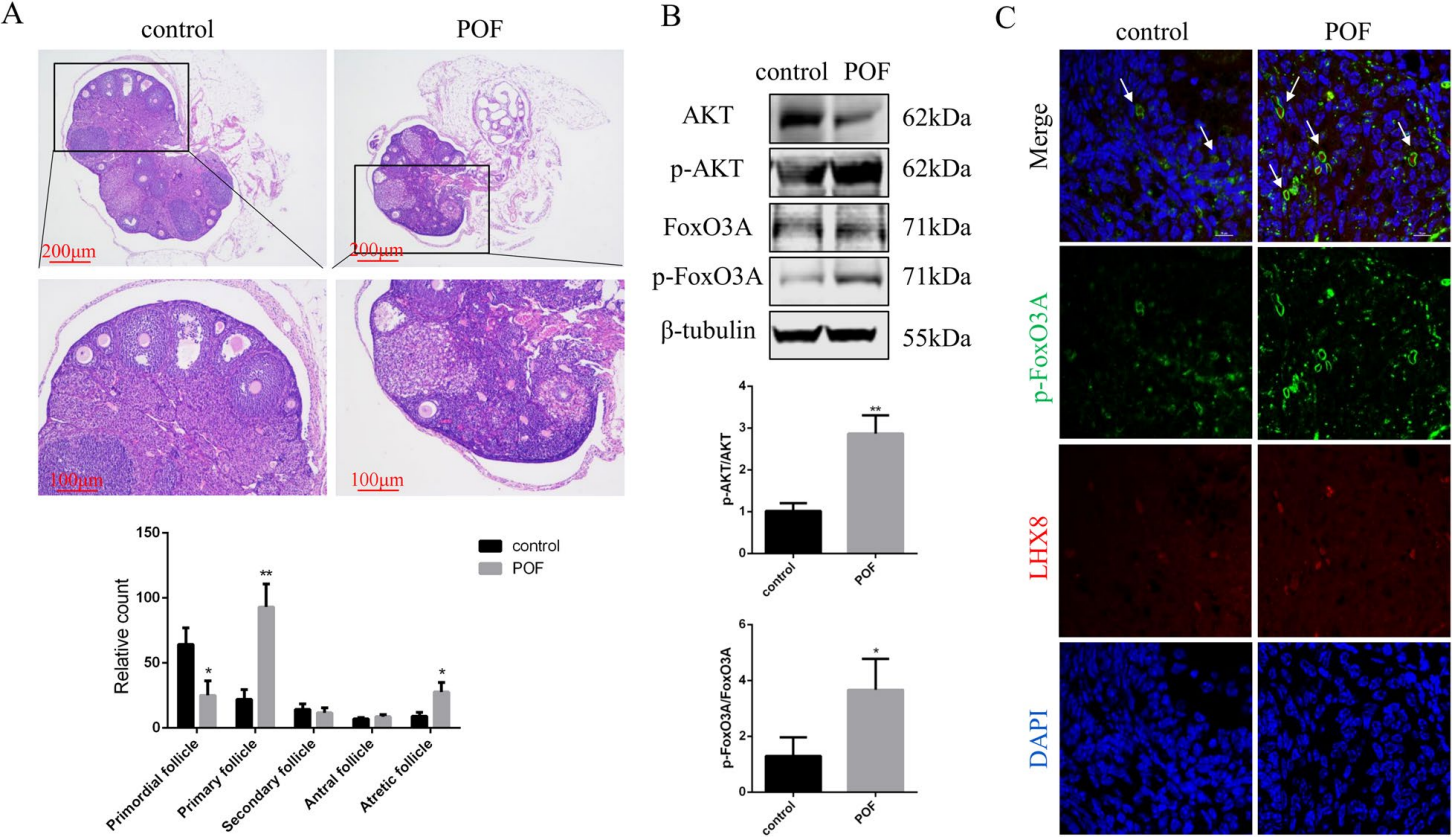
Effects of cisplatin treatment on ovarian follicle activation in mice. **A** H&E staining displaying ovarian morphology and columns representing the proportion of follicles at various stages of development between the control group and POF group. **B** Protein levels of AKT, p-AKT, FoxO3A, and p-FoxO3A in the ovaries of the two groups. **C** Images of immunofluorescence showing the localization of p-FoxO3A using anti-p-FoxO3A antibodies (green) in the two groups. The primordial follicles were detected using an anti-LHX8 antibody (red). All nuclei were stained with DAPI. Control group, *n* = 10; POF group, *n* = 10. H&E, hematoxylin and erosion; POF, premature ovarian failure; DAPI, 4′6-diamidino-2-phenylindole. *P < 0.05; **P < 0.01, two-tailed Student’s *t* test. Scale bars represent 200 μm, 100 μm (**A**), and 10 μm (**C**)

To further evaluate the ovarian reserve of each group, the ovaries in the two groups were collected for histological analysis and the number of follicles at various stages of development was counted between the two groups. Notably, the ovaries in the POF group appeared to be atrophic, containing fewer healthy follicles compared with those in the control group. The POF ovaries exhibited a significant decrease in the number of primordial follicles and a dramatic increase in the number of primary follicles and atretic follicles after chemotherapy compared with those in the control ovaries (Fig. 3A). Previous studies have reported that primordial follicle activation was a causative factor accelerating follicular depletion and atresia governed by the PI3K/AKT pathway [17–19]; therefore, we detected the levels of related signaling molecules in the control and POF mice. Western blotting results showed increased levels of phosphorylated AKT (p-AKT) and FoxO3A (p-FoxO3A) in the ovaries of POF group compared with those in the control group (Fig. 3B). FoxO3A is phosphorylated and exported from the nucleus to the cytoplasm during primordial follicle activation, thus, the cytosolic localization of p-FoxO3A was evaluated by immunofluorescence analysis. LIM Homeobox 8 (LHX8) is known as germ-cell specific marker; therefore, the nuclear export of p-FoxO3A was analyzed by immunofluorescence using anti-LHX8 and anti-p-FoxO3A antibodies. The results showed increased phosphorylation and nuclear shuttling of FoxO3A in the ovaries of cisplatin-treated mice (Fig. 3C). Collectively, our results suggested that cisplatin treatment resulted in the increased phosphorylation of PI3K/AKT pathway components, leading to the activation of this signaling pathway and increased nuclear export of FoxO3A, thus inducing abnormal activation of primordial follicles within ovarian tissue and causing dysfunction in POF mice.

### miR-144-3p is downregulated in GCs of POF mice

**Fig. 4:**
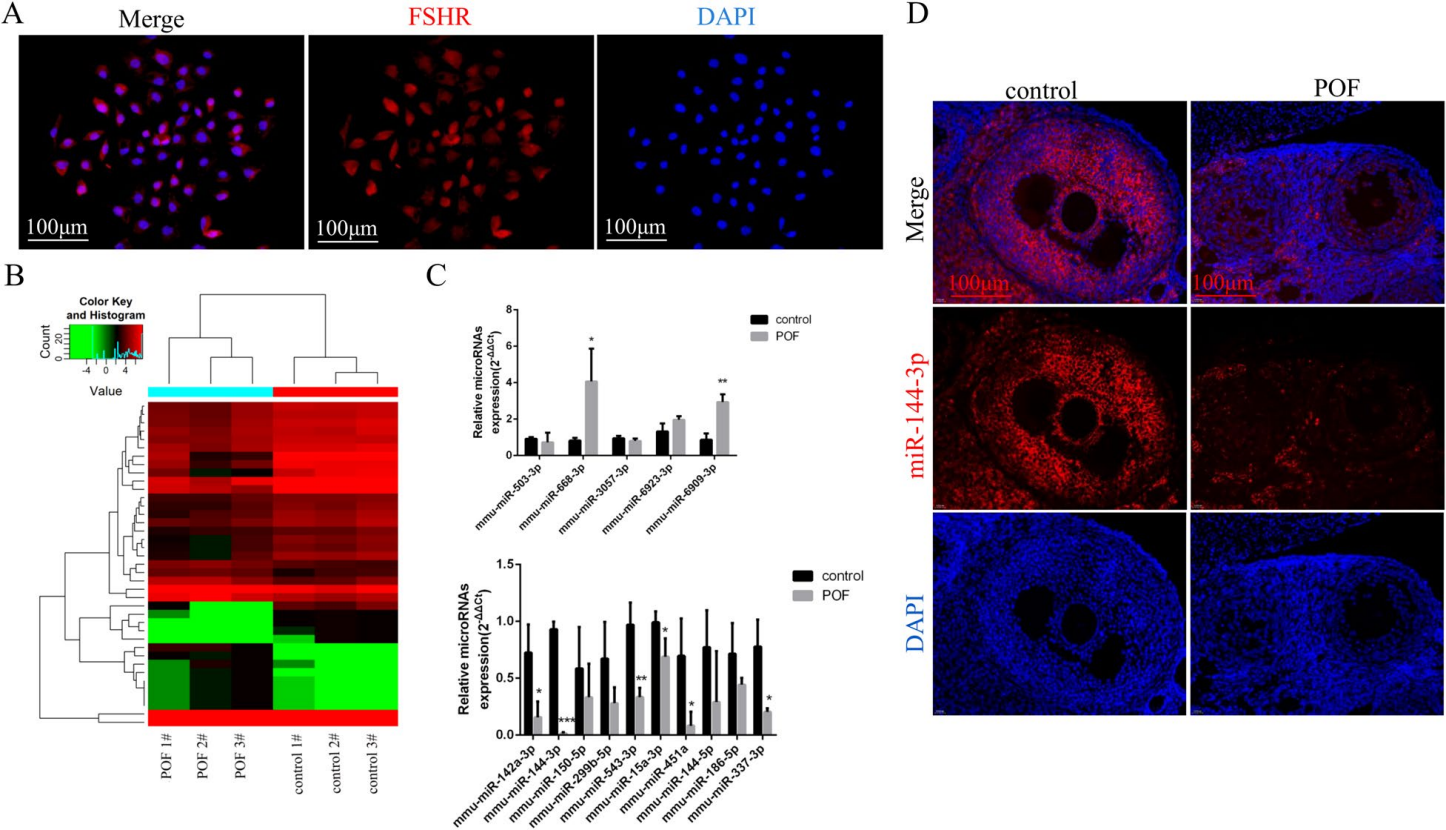
miRNA microarray analysis was performed to detect differentially expressed miRNAs in GCs of mice. **A** Identification of GCs. Isolated GCs show a spindle-shaped morphology. The red staining indicates FSHR-positive GCs. The blue DAPI stain indicates the GC nuclei. **B** miRNA expression in the POF group and the control group from three biological replicate samples was profiled using microRNA array. **C** Real-time PCR was used to verify the microarray results. **D** Fluorescence in situ hybridization detected expression of miR-144-3p in the control group and POF group. Control group, *n* = 10; POF group, *n* = 10. GCs: granulosa cells; FSHR: follicle-stimulating hormone receptor; DAPI: 4′6-diamidino-2-phenylindole; POF: premature ovarian failure. *P < 0.05; **P < 0.01; *** *P* < 0.001, two-tailed Student’s *t* test. Scale bar, 100 μm

GCs were isolated from ovarian tissues. After FSHR immunostaining, a GC-specific marker, the cells stained red were identified as GCs (Fig. 4A). To explore aberrantly expressed miRNAs and genes in GCs from POF mice, we first conducted miRNA and mRNA microarrays analysis of GCs from the POF group and the control group. A total of 39 miRNAs (fold change > 2, *P* < 0.05) and 1493 mRNAs (fold change > 2, *P* < 0.05) were identified to be differentially expressed between the two groups (Fig. 4B). Of these 39 differentially expressed miRNAs, 14 were upregulated and 25 were downregulated. To validate the microarray results, we analyzed miRNA expression using qRT-PCR in GCs from the POF and control groups (Fig. 4C). Among these candidates, miR-144-3p was significant downregulated in POF mice. Accordingly, compared with that in the control group, there was a significant decrease in the expression level of miR-144-3p in the POF ovaries, as detected using Fluorescence in situ hybridization analysis (Fig. 4D).

### *Map3k9* is upregulated in GCs of POF mice

**Fig. 5:**
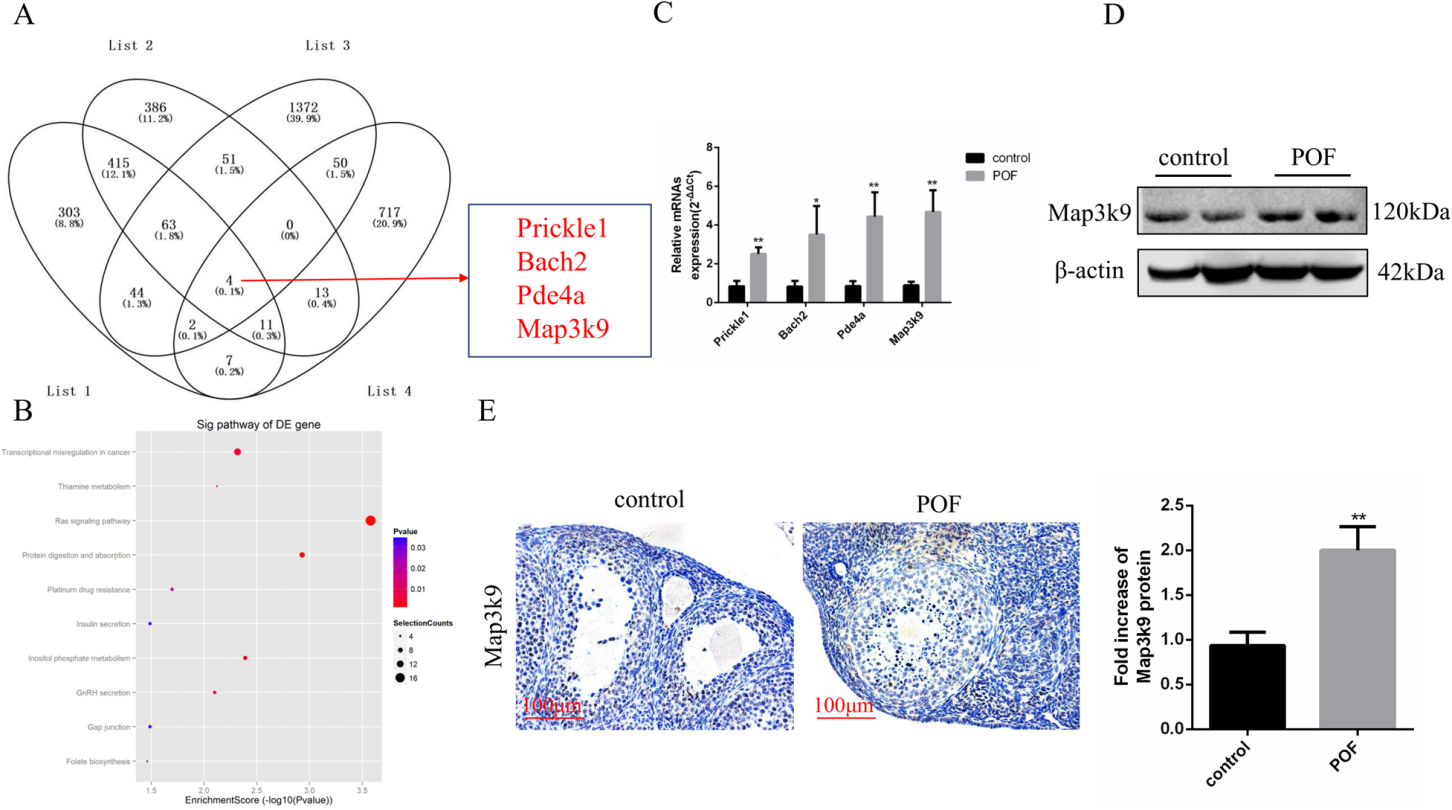
*Map3k9* is upregulated in GCs of POF mice. **A** Bioinformatics prediction combined with mRNA microarray analysis were performed to identify the potential target genes of miR-144-3p. **B** Differentially expressed gene pathway enrichment analysis in the POF group and the control group. **C** Real-time PCR was used to evaluate the relative expression of target genes of miR-144-3p in the POF group and the control group. **D** Protein levels of MAP3K9 in both groups were detected using western blotting.** E** Immunohistochemistry analysis of the protein expression of MAP3K9 in both groups. Control group, *n* = 10; POF group, *n* = 10. GCs: granulosa cells; POF: premature ovarian failure. *P < 0.05; **P < 0.01, two-tailed Student’s *t* test. Scale bar, 100 μm

To identify the mechanism by which the cisplatin-induced downregulation of miR-144-3p leads to POF, we analyzed the potential target genes of miR-144-3p. Three databases, TargetScan7.2, RNA22 and miRDB, combined with mRNA microarray analysis, were used to search for the genes potentially targeted by miR-144-3p. Four potential target genes (*Prickle1*, *Bach2*, *Pde4a*, and *Map3k9*) of miR-144-3p were identified (Fig. 5A). The differentially expressed gene pathway enrichment analysis between the POF group and control group is shown in Fig. 5B. The results of qRT-PCR confirmed that the four candidate genes of miR-144-3p displayed increased expression in the ovarian GCs of POF mice. In particular, the increase in *Map3k9* expression was the most prominent (Fig. 5C). Accordingly, compared with that in the control group, there was a significant increase in the protein level of MAP3K9 in the POF ovaries, as detected using western blotting (Fig. 5D) and immunohistochemistry analysis (Fig. 5E). Therefore, *Map3k9* was selected as the follow-up study object.

### *MAP3K9* is a target of miR-144-3p

**Fig. 6:**
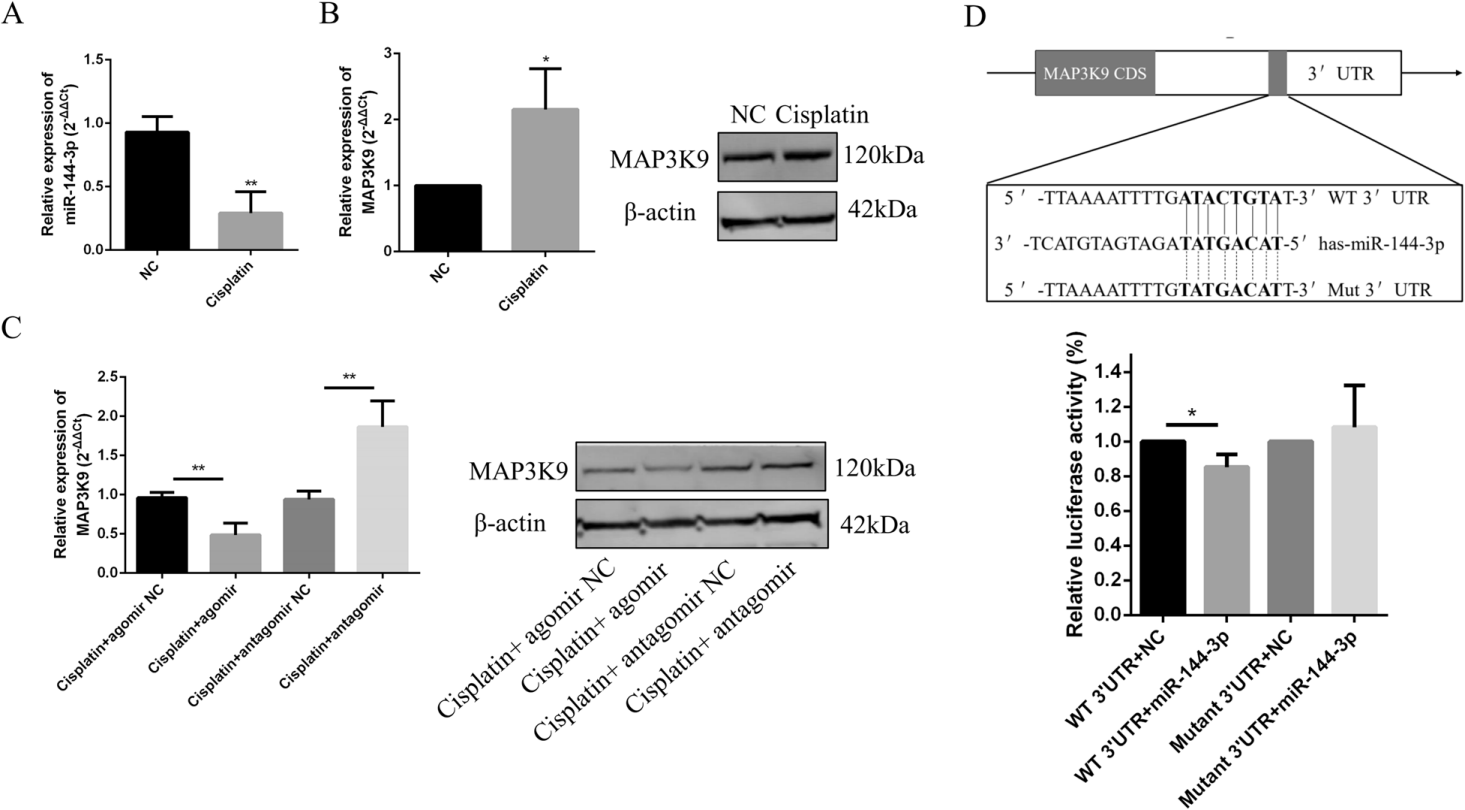
*MAP3K9* is a target of miR-144-3p.** A** Real-time PCR showing that miR-144-3p expression was significantly decreased in cisplatin injured COV434 cells.** B** MAP3K9 mRNA and protein levels increased significantly, as analyzed using real-time PCR and western blotting, respectively.** C** Real-time PCR and western blotting were performed to detect the mRNA and protein levels of MAP3K9 in the injured COV434 cells transfected with miR-144-3p agomir and antagomir. **D** Predicted target sites and relevant mutation sequences are shown. Luciferase reporter assay results demonstrating that overexpression of miR-144-3p could reduce the site-specific luciferase activity of the *MAP3K9* gene with wild type 3′ untranslated region (UTR). *P < 0.05; **P < 0.01, two-tailed Student’s *t* test

GCs are important component of follicles that are susceptible to chemotherapy-induced damage. Herein, a human ovarian granulosa tumor-derived cell line (COV434) was used. To further investigate the regulation of miR-144-3p on *MAP3K9* expression and GCs function in vitro, we first established a cisplatin (4 mg/l)-injured COV434 cell model. Consistent with the microarray results, qRT-PCR showed that the miR-144-3p expression level was significantly decreased in cisplatin-damaged COV434 cells (Fig. 6A), while MAP3K9 expression was demonstrated to be increased significantly increased at the protein mRNA levels (Fig. 6B). Then, we performed qRT-PCR and western blotting to examine the expression of MAP3K9 in cisplatin-treated COV434 cells treated with the miR-144-3p agomir or antagomir. Notably, MAP3K9 mRNA and protein levels were significantly downregulated by the overexpression of miR-144-3p, but were enhanced when miR-144-3p expression was inhibited in cisplatin-injured COV434 cells (Fig. 6C), indicating the negative regulation of miR-144-3p on *MAP3K9* expression. To confirm the predicted binding site of miR-144-3p in the 3′ UTR of *MAP3K9*, we cloned the wild-type and mutated 3′ UTR of *MAP3K9* into a luciferase reporter vector. The results of the dual-luciferase reporter assay showed that miR-144-3p overexpression suppressed the relative activity of the luciferase reporter harboring the wild-type 3′ UTR of *MAP3K9*, while luciferase activity levels of the mutated 3′ UTR were unchanged (Fig. 6D). Our results showed that *MAP3K9* is a target of miR-144-3p.

### miR-144-3p targets *MAP3K9* to regulate the apoptosis of GCs via the p38 MAPK pathway

**Fig. 7:**
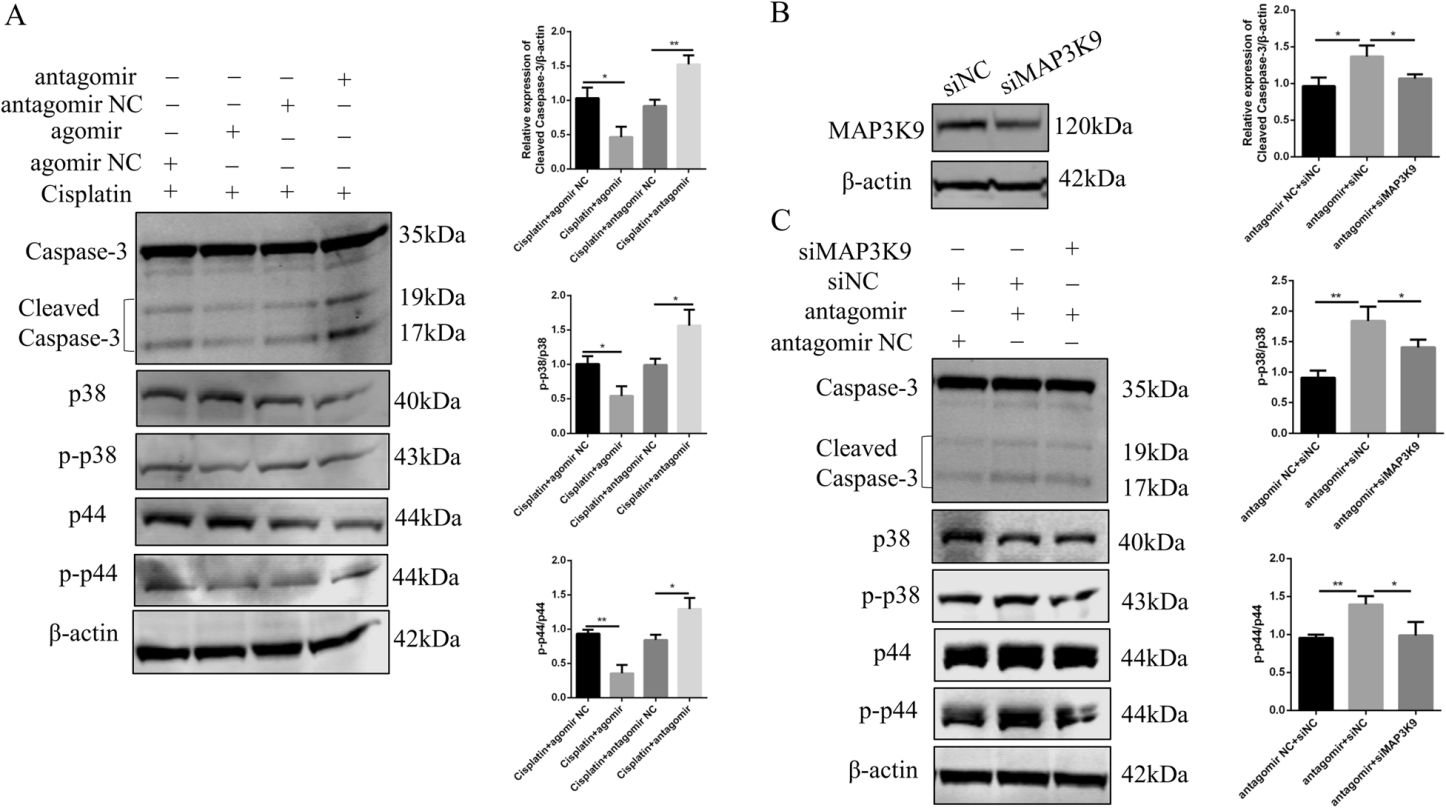
miR-144-3p protects GCs against cisplatin-induced apoptosis by targeting MAP3K9 via the p38 MAPK pathway. **A** Western blotting to detect the protein levels of cleaved caspase-3 and p38 MAPK pathway-related molecules in cisplatin injured COV434 cells. **B** Western blotting to determine the level of MAP3K9 in COV434 cells transfected with *MAP3K9* siRNA and scrambled control oligonucleotide. **C** Cleaved caspase-3 and p38 MAPK pathway-related proteins levels were analyzed by western blotting in miR-144-3p antagomir and *MAP3K9* siRNA co-treated COV434 cells. GCs, granulosa cells. *P < 0.05; **P < 0.01, two-tailed Student’s *t* test

Next, we determined the impact of miR-144-3p on chemotherapy-induced GC apoptosis in vitro. Western blotting was performed to detect the protein levels of cleaved caspase-3 in the cisplatin-injured COV434 cells transfected with the miR-144-3p agomir and antagomir. As shown in Fig. 7A, when miR-144-3p was overexpressed in injured COV434 cells, the protein level of cleaved caspase-3 decreased, whereas inhibition of miR-144-3p expression markedly increased the protein level of cleaved caspase-3. This result demonstrated that miR-144-3p would have an inhibitory effect on chemotherapy-induced GC apoptosis.

Our functional experiments demonstrated that miR-144-3p regulated *MAP3K9* expression and GCs apoptosis negatively. MAP3K9 is an upstream activator of the p38 MAPK signaling pathway. To determine the molecular mechanism underlying the enhanced GC apoptosis in cisplatin-treated COV434 cells, we studied the members of p38 MAPK pathway (p38, p–p38, p44, and p–p44). In miR-144-3p overexpressing cells, the levels of p–p38 and p–p44 were significantly reduced; in contrast, miR-144-3p downregulation increased the p–p38 and p–p44 levels (Fig. 7A). miR-144-3p can markedly repress *MAP3K9*; therefore, we further assessed whether MAP3K9 can mediate the observed biological effects of miR-144-3p. We first silenced *MAP3K9* in COV434 cells using a specific siRNA. Western blotting confirmed the efficient decrease in the level of MAP3K9 in COV434 cells at 48 h post transfection (Fig. 7B). For the cell apoptosis analysis, cleaved caspase-3 expression was detected using western blotting. The results showed that the cleaved caspase-3 level was much higher in COV434 cells transfected with miR-144-3p antagomir than in those transfected with the negative control, while *MAP3K9* silencing partially reversed the increased level of cleaved caspase-3 induced by the miR-144-3p antagomir in COV434 cells (Fig. 7C). Next, we detected p38 MAPK pathway-related molecules in the above cells. Cells treated with the miR-144-3p antagomir displayed dramatically increased p–p38 and p–p44 levels, while *MAP3K9* silencing manifested the opposite results (Fig. 7C). Taken together, miR-144-3p overexpression caused decreased GC apoptosis by suppressing the downstream p38 MAPK signaling pathway, whereas reduced expression of miR-144-3p accelerated cell apoptosis through phosphorylation and activation of p38 MAPK pathway members. Importantly, suppression of MAP3K9 partially rescued miR-144-3p downregulation-induced GC damage via inhibition of the phosphorylation of p38 MAPK pathway components.

### miR-144-3p partially prevents cisplatin-induced primordial follicle activation via MAP3K9 in mouse ovaries in vivo

**Fig. 8:**
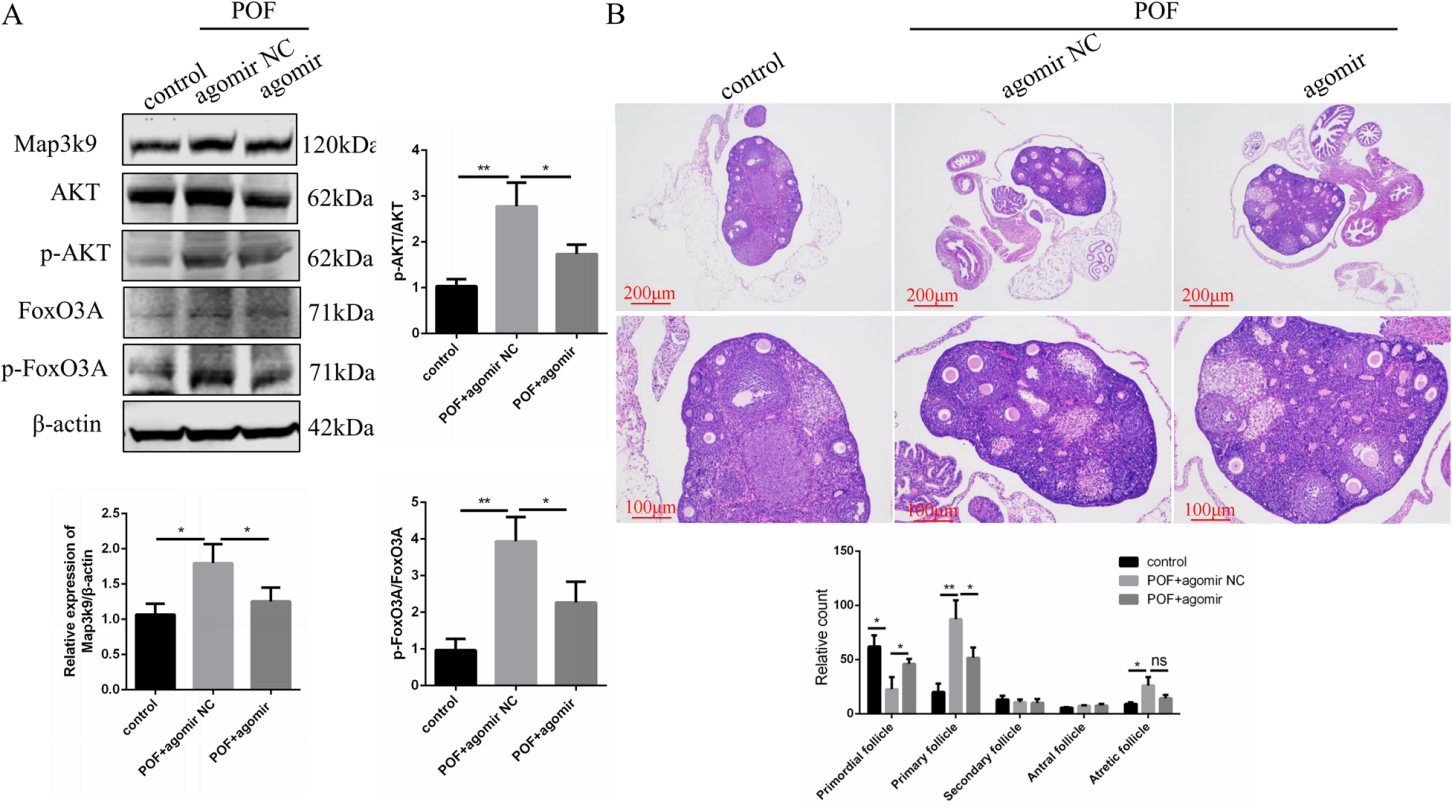
miR-144-3p attenuates cisplatin-induced primordial follicle activation via MAP3K9 in mouse ovaries in vivo. **A** Western blotting detection of MAP3K9 and PI3K/AKT pathway-related proteins in mice. **B** H&E staining displaying ovarian morphology and columns representing the proportion of follicles at various stages of development in mice. Control group, *n* = 3; POF + agomir NC group, *n* = 3; POF + agomir group, *n* = 3. H&E: hematoxylin and erosion; POF: premature ovarian failure. *P < 0.05; **P < 0.01, two-tailed Student’s *t* test

To further understand the detailed mechanisms underlying the alteration in primordial follicle activation following cisplatin treatment, we analyzed the expression of signaling molecules involved in the PI3K/AKT pathway in the ovaries of mice following cisplatin treatment, with or without miR-144-3p agomir treatment. After intraperitoneal injection of cisplatin for 7 days, high MAP3K9 levels were observed in the cisplatin alone treatment group, whereas MAP3K9 levels were remarkably repressed in the cisplatin and miR-144-3p agomir co-treatment group, indicating the negative regulation of miR-144-3p on *Map3k9* expression in vivo (Fig. 8A). Western blotting demonstrated a remarkable increase of p-AKT and p-FoxO3A levels in the POF group compared with those in the control group. However, overexpression of miR-144-3p significantly decreased p-AKT and p-FoxO3A protein levels in mice receiving miR-144-3p agomir concurrently with cisplatin (Fig. 8A). In addition, a significant increase in the number of primordial follicles and a dramatic decrease in the number of primary follicles were observed after miR-144-3p agomir injection (Fig. 8B). Therefore, our results suggest that cisplatin-induced premature primordial follicle activation is mainly associated with the phosphorylation and stimulation of PI3K/AKT pathway components, resulting in the exhaustion of the primordial follicle pool in the ovary. Furthermore, miR-144-3p prevents cisplatin-induced primordial follicle disruption by blocking the phosphorylation of PI3K/AKT pathway members via downregulation of *Map3k9*.

## Discussion

Anticancer treatment is a well-known risk factor for POF. With the wide application of chemotherapeutic agents in various types of tumors and immune diseases, chemotherapy-induced POF has received increased attention among all the causes of POF. Detailed information focusing on the detrimental effects of chemotherapy exposure on ovarian function has been published [20, 21]. It is generally believed that chemotherapeutic agents can damage the ovary by inducing prenatal loss of oogonia, direct loss of primordial follicles, accelerated primordial follicle activation, follicular atresia, and damage to the ovarian stroma and the microvascular architecture [21, 22]. As one of the most commonly used anticancer drugs, cisplatin can cause POF by triggering the activation of primordial follicles and increasing the atresia of ovarian follicles [21].

GC apoptosis is confirmed to be involved in the development of follicular atresia and follicle loss [23]. Follicular activation and growth are closely related to the proliferation and anti-apoptosis in granulosa cells. The somatic primordial follicle granulosa cells initiate the activation of primordial follicles and govern the quiescence or awakening of dormant oocytes. Activation of mTORC1 signaling in granulosa cells of primordial follicles accelerates the differentiation of primordial follicle granulosa cells into granulosa cells and cause premature activation of all dormant oocytes and primordial follicles [24]. Primordial follicle granulosa cells trigger the awakening of dormant oocytes through KIT ligand, and the essential communication network between the somatic cells and germ cells is based on signaling between the mTORC1–KITL cascade in primordial follicle granulosa cells and KIT–PI3K signaling in oocytes [25]. Many growth factors have been reported to be functional in regulating the activation of primordial follicles in vitro, including leukemia inhibitory factor, basic fibroblast growth factor and platelet-derived growth factor [17]. Granulosa cell-derived C-type natriuretic factor not only suppresses the final maturation of oocytes to undergo germinal vesicle breakdown before ovulation but also promotes preantral and antral follicle growth. In addition, several oocyte- and granulosa cell-derived factors stimulate preantral follicle growth by acting through wingless, receptor tyrosine kinase, receptor serine kinase, and other signaling pathways [26]. Oocyte–GC bidirectional communications via signal transduction or direct cell-to-cell contact provide the molecular and structural basis for effective oocyte–GC crosstalk, which is required for adequate follicular growth and maturation [27]. In this study, we illustrated enhanced GC apoptosis and accelerated primordial follicle activation in a cisplatin-induced mouse POF model. This might accelerate follicular apoptosis and follicle reservoir utilization via multiple molecular reactions. A better understanding of the molecular mechanisms underlying cisplatin-mediated ovarian dysfunction might allow the development of efficient and targeted treatments for patients diagnosed with POF and for infertile women of advanced reproductive age.

Recently, miRNAs have been recognized to play important regulatory roles in ovarian function [23, 28] and miRNAs are indicated to be involved in POF pathogenesis. Zhang et al. discovered the harmful effects of miR-127-5p on proliferation and DNA repair function of GCs via the *HMGB2* gene (encoding high mobility group box 2) and its predictive value in POF [29]. Moreover, miR-145 was reported to protect GCs against oxidative stress-induced apoptosis by targeting *KLF4* (encoding Kruppel-like factor 4), thereby preventing abnormal follicular atresia and improving the outcomes of ovarian dysfunction [30]. It has been found that miR-146b-5p overexpression ameliorates POF in mice by inhibiting the DAB2IP/ASK1/p38-MAPK pathway and γH2A.X phosphorylation [31]. In the current study, we performed miRNA and mRNA microarray screening on GCs derived from cisplatin-induced POF mice and identified significantly downregulated miR-144-3p in GCs from POF mice. Previous research has reported that the expression of miR-144-3p was greatly reduced in patients with polycystic ovarian syndrome (PCOS) and PCOS rat models. Meanwhile, miR-144-3p overexpression could induce ovarian GCs growth and repress cell apoptosis by targeting *HSP70* (encoding heat shock protein 70), which might function as a novel target for PCOS treatment [32]. We hypothesized that miR-144-3p might also be involved in the regulation of ovarian function in POF. The findings of this study supported our hypothesis and revealed that miR-144-3p alleviated cisplatin-induced GC damage and primordial follicle activation by repression its downstream target gene, *MAP3K9*, which sheds light on the epigenetic mechanism involved in the pathogenicity of POF.

MAP3K9 is an upstream activator of the p38 MAPK signaling pathway. p38 MAPK pathway members function in a variety of cellular processes, including cell growth, proliferation, differentiation, migration, and apoptosis [33]. Several studies have demonstrated the clear involvement of the p38 MAPK pathway in the response to treatment with chemotherapeutic agents [34–36]. This study explored the correlation between the pathway and GC apoptosis, demonstrating that miR-144-3p overexpression decreased GC cell apoptosis resulting from suppression of the downstream p38 MAPK pathway, while reduced expression of miR-144-3p manifested the opposite results through phosphorylation and activation of p38 MAPK pathway members. Furthermore, *MAP3K9* downregulation abrogated the miR-144-3p inhibition-induced effects partially. These results suggested that miR-144-3p prevents GC cell apoptosis by repressing the p38 MAPK pathway via negatively regulating *MAP3K9*.

Activation of dormant primordial follicles is the first step in follicular development and is fundamental to determine the ovarian reserve of females [37]. Although the exact factors involved in primordial follicle recruitment and growth have yet to be elucidated, the PI3K/phosphatase and tensin homolog (PTEN) signaling pathway has been reported to be the key controller for follicular activation [38]. In the present study, our results suggested that during the process of primordial follicle initiation in vivo, the addition of miR-144-3p to cisplatin-treated ovaries is accompanied by decreased expression of its target gene, *Map3k9*, and suppression of the downstream PI3K/AKT signaling pathway. Therefore, miR-144-3p has a protective effect on cisplatin-induced abnormal activation of primordial follicles by repressing the PI3K/AKT cascade via downregulating *Map3k9.*

In conclusion, our data revealed that miR-144-3p significantly downregulated in GCs from POF mice. The in vitro and in vivo experiments revealed that miR-144-3p can protect against cisplatin induced GC cell apoptosis and abnormal activation of primordial follicles via the p38 MAPK and PI3K/AKT pathways, respectively, by directly targeting the *MAP3K9* gene. This study clarified the biological effects of miR-144-3p and its detailed molecular mechanisms responsible for POF progression and suggested new strategies for POF management.

### Supplementary Information

**Additional file 1: Table S1.** Primers used for real-time PCR.

## Data Availability

The data sets used or analyzed during the current study are available from the corresponding author on reasonable request.
