# Supplementary material for: MicroRNA-144-3p protects against chemotherapy-induced apoptosis of ovarian granulosa cells and activation of primordial follicles by targeting MAP3K9
Source: Eur J Med Res. 2023 Aug 3;28:264. doi: 10.1186/s40001-023-01231-2 (PMC10399062; doi:10.1186/s40001-023-01231-2)
Supplement: Supplementary file 1 — Additional file 1: Table S1. Primers used for real-time PCR. [file 40001_2023_1231_MOESM1_ESM.docx]

**Table S1 Primers used for Real-time PCR**

| Primer names |  | Sequences |
| --- | --- | --- |
| mmu-miR-543-3p |  | 5＇-CCAAACATTCGCGGTGCACTTCTT-3＇ |
| mmu-miR-451a |  | 5＇-CGCGAAACCGTTACCATTACTGAGTT-3＇ |
| mmu-miR-337-3p |  | 5＇-CCGCTCAGCTCCTATATGATGCCTTT-3＇ |
| mmu-miR-299b-5p |  | 5＇-GCGGTTTACCGTCCCACATACAT-3＇ |
| mmu-miR-186-5p |  | 5＇-CGCCAAAGAATTCTCCTTTTGGGCT-3＇ |
| mmu-miR-15a-3p |  | 5＇-CAGGCCATACTGTGCTGCCT-3＇ |
| mmu-miR-150-5p |  | 5＇-TCTCCCAACCCTTGTACCAGTG-3＇ |
| mmu-miR-144-5p |  | 5＇-CGCGCGCGGATATCATCATATACTGTA-3＇ |
| mmu-miR-144-3p |  | 5＇-CGCGCGCTACAGTATAGATGATGTACT-3＇ |
| mmu-miR-142a-3p |  | 5＇-CGCCGTGTAGTGTTTCCTACTTTATGG-3＇ |
| mmu-miR-6923-3p |  | 5＇-ACACTCCCTCCTCCTCCC-3＇ |
| mmu-miR-6909-3p |  | 5＇-TATGCCTTCCCCGGCCTC-3＇ |
| mmu-miR-668-3p |  | 5＇-TATGTCACTCGGCTCGGCC-3＇ |
| mmu-miR-503-3p |  | 5＇-CGGAGTATTGTTTCCACTGCCTGG-3＇ |
| mmu-miR-3057-3p |  | 5＇-TATCCCACAGGCCCAGCTC-3＇ |
| mouse-U6-F |  | 5＇-GCTTCGGCAGCACATATACTAAAAT-3＇ |
| mouse-U6-R |  | 5＇-CGCTTCACGAATTTGCGTGTCAT-3＇ |
| has-miR-144-3p |  | 5＇-CGCGAGCGCTACAGTATAGATGATGT -3＇ |
| has-U6 |  | 5＇- AGAGAAGATTAGCATGGCCCCTG -3＇ |
| mouse-Map3k9-F |  | 5＇-AGGAGAAGAAGAGGCGTGAAGGTC-3＇ |
| mouse-Map3k9-R |  | 5＇-AGCAGTGTCAAGGAGGCAGAGG-3＇ |
| mouse-Pde4a-F |  | 5＇-ACTCTCCTGGCTCCACATGATACC-3＇ |
| mouse-Pde4a-R |  | 5＇-GGCTGTCTCCTGCTTCAAACTCTC-3＇ |
| mouse-Bach2-F |  | 5＇-CGGAAACTGGTGTGCGAGAAGG-3＇ |
| mouse-Bach2-R |  | 5＇-TGGATCTGCTCTGGACTCTGGATG-3＇ |
| mouse-Prickle1-F |  | 5＇-ACATCCACGCCTCTGACTCCTC-3＇ |
| mouse-Prickle1-R |  | 5＇-CAAGAGAAGAGACTGTCGGCACTG-3＇ |
| mouse-β-actin-F |  | 5＇-TTCTTTGCAGCTCCTTCGT-3＇ |
| mouse-β-actin-R |  | 5＇-TTCTGACCCATTCCCACC-3＇ |
| human-MAP3K9-F |  | 5＇-TGCCCTAAGAGGGGTATGTCT-3＇ |
| human-MAP3K9-R |  | 5＇-CACTCTATTCAAAGGTCCTCCAC-3＇ |
| human-β-actin-F |  | 5＇-CTGGTGCCTGGGGCG-3＇ |
| human-β-actin-R |  | 5＇-AGCCTCGCCTTTGCCGA-3＇ |
